# Herbal Medicine Formulas for Parkinson's Disease: A Systematic Review and Meta-Analysis of Randomized Double-Blind Placebo-Controlled Clinical Trials

**DOI:** 10.3389/fnagi.2018.00349

**Published:** 2018-11-08

**Authors:** Chun-Shuo Shan, Hong-Feng Zhang, Qing-Qing Xu, Yi-Hua Shi, Yong Wang, Yan Li, Yan Lin, Guo-Qing Zheng

**Affiliations:** Department of Neurology, The Second Affiliated Hospital and Yuying Children's Hospital of Wenzhou Medical University, Wenzhou, China

**Keywords:** Parkinson's disease, randomized double-blind placebo-controlled clinical trial, traditional Chinese medicine, meta-analysis, systematic review

## Abstract

**Background:** Parkinson's disease (PD) is a debitlitating, chronic, progressive neurodegenerative disorder without modifying therapy. Here, we aimed to evaluate the available evidence of herbal medicine (HM) formulas for patients with PD according to randomized double-blind placebo-controlled clinical trials.

**Methods:** HM formulas for PD were searched in eight main databases from their inception to February 2018. The methodological quality was assessed using Cochrane Collaboration risk of bias tool. Meta-analysis was performed using RevMan 5.3 software.

**Results:** Fourteen trials with Seventeen comparisons comprising 1,311 patients were identified. Compared with placebo groups, HM paratherapy (*n* = 16 comparisons) showed significant better effects in the assessments of total Unified Parkinson's Disease Rating Scale (UPDRS) (WMD: −5.43, 95% CI:−8.01 to −2.86; *P* < 0.0001), UPDRS I (WMD: −0.30, 95% CI: −0.54 to −0.06; *P* = 0.02), UPDRS II (WMD: −2.21, 95% CI: −3.19 to −1.22; *P* < 0.0001), UPDRS III (WMD: −3.26, 95% CI:−4.36 to −2.16; *P* < 0.00001), Parkinson's Disease Quality of Life Questionnaire (*p* < 0.01) and Parkinson's Disease Questionnaire-39 (WMD: −7.65, 95% CI: −11.46 to −3.83; *p* < 0.0001), Non-motor Symptoms Questionnaire (*p* < 0.01) and Non-Motor Symptoms Scale (WMD: −9.19, 95% CI: −13.11 to −5.28; *P* < 0.00001), Parkinson's Disease Sleep Scale (WMD: 10.69, 95% CI: 8.86 to 12.53; *P* < 0.00001), and Hamilton depression rating scale (WMD: −5.87, 95% CI: −7.06 to −4.68; *P* < 0.00001). The efficiency of HM monotherapy (*n* = 1 comparison) was not superior to the placebo according to UPDRS II, UPDRS III and total UPDRS score in PD patients who never received levodopa treatment, all *P* > 0.05. HM formulas paratherapy were generally safe and well tolerated for PD patients (RR: 0.41, 95% CI: 0.21 to 0.80; *P* = 0.009).

**Conclusion:** The findings of present study supported the complementary use of HM paratherapy for PD patients, whereas the question on the efficacy of HM monotherapy in alleviating PD symptoms is still open.

## Introduction

Parkinson's disease (PD) is a common chronic neurodegenerative disease characterized by the degeneration of dopaminergic neurons in the substantia nigra (SN) (Kalia and Lang, 2015), and presents with non-motor or/and motor syndrome (Rogers et al., 2017). In the Global Burden of Diseases, Injuries, and Risk Factors Study (GBD) 2016, PD was the second leading cause in neurological disorders of years lived with disability (YLDs), contributing to 6.1 million of patients (GBD, 2016 Disease and Injury Incidence and Prevalence Collaborators, 2017). From 2005 to 2015, global deaths due to PD increased by 42.4%, to 117.4 thousands deaths (GBD, 2015 Mortality and Causes of Death Collaborators, 2016), as a result of population aging. With the growing incidence, PD seriously hurt the physical and mental health of the elderly, also produced a heavy economic burden on both families and society. The average annual cost per PD patient was $22,800 in the United States (Kowal et al., 2013) and $36,085 in the UK (Findley et al., 2011). Current conventional treatment for PD is based on the dopamine (DA) replacement therapies and reduction of DA degradation, including levodopa, DA receptor agonists, monoamine oxidase-B inhibitors, catechol-O-methyltransferase inhibitors and other types of drugs (Rogers et al., 2017). However, all the current therapeutic approaches remain palliative and can't inhibit or reverse the progression of PD (Athauda and Foltynie, 2015). Furthermore, frequently with these treatments can lead to obvious adverse events and efficacies diminished, as well as induce therapy-related motor complications such as dyskinesia, choreoathetosis, and fluctuations in motor function (Jenner, 2015). A safer and more effective alternative treatment of PD is increasingly demanded.

The therapy of herbal medicine (HM) for PD is particularly common. In China, HM could be traced in the Huangdi Neijing (Inner Canon of Yellow Emperor) (Zheng, 2009), the earliest existing classics in Chinese herbal medicine (CHM). Up to now, HM is still very popular in the treatment of PD especially in Asian countries (Wang et al., 2011, 2013). Previous reviews (Wang et al., 2012; Zhang et al., 2015) found lack of evidence of supporting the use of HM for PD patients because of the generally low-quality studies included. Here, we performed a systematic review and meta-analysis of randomized double-blind placebo-controlled clinical trials of HM formulas for PD patients and further explored the mechanisms of high-frequently used herbs against PD.

## Methods

This systematic review and meta-analysis is conducted according to the Preferred Reporting Items for Systematic Reviews and Meta-Analyses: The PRISMA Statement (Moher et al., 2010b) and our previous study (Yang et al., 2017).

### Search strategy

Randomized double-blind placebo-controlled clinical trials of HM formulas for PD were searched in eight databases from their inception to February 2018. They are PubMed, EMBASE, Cochrane Central Register of Controlled Trials (CENTRAL), Web of science, Chinese National Knowledge Infrastructure (CNKI), Chinese VIP Information, Wanfang database and Chinese Biological Medical Literature Database (CBM). Moreover, we hand searched additional relevant studies using the reference list of previous reviews. The search strategy of PubMed was as follows, and was modified to suit other English or Chinese databases.

PubMed search strategy:

#1. Parkinson disease [mh]
#2. Parkinson^*^[tiab]
#3. #1OR #2
#4. Medicine, Chinese Traditional [mh]
#5. Herbal Medicine [mh]
#6. Integrative Medicine [mh]
#7. traditional Chinese medicine [tiab]
#8. herb^*^ [tiab]
#9. #4 OR #5 OR #6 OR #7 OR #8
#10. #3 And #9

### Study selection

Two authors (CS-S and H-FZ) independently engaged in the selection of studies by reading study titles, abstracts and full texts. The disagreement was resolved by the corresponding author (GZ) or repeated discussion.

#### Inclusion criteria

Type of study: the articles were randomized double-blind placebo-controlled clinical trials.

Type of participants: participants were of any age or sex with a confirmed diagnosis of PD according to the UK Brain Bank criteria (Hughes et al., 1992), Chinese National Diagnosis Standard (CNDS) for PD in 1984 (Wang, 1985), CNDS updated version in 2006 for PD (Zhang, 2006) or other formal comparable criteria.
Type of intervention: Analyzed interventions were HM formulas or HM formulas plus western conventional medicine (WCM) according to PD treatment guidelines, ^2^ regardless of the form of the drug, dosage, frequency or duration of the treatment. Comparator interventions were placebo or placebo plus WCM.
Type of outcome measures: the primary outcomes were total Unified Parkinson's Disease Rating Scale (UPDRS) score, UPDRS I (Mental Score), UPDRS II (Activities of Daily Life), UPDRS III (Motor Score), and UPDRS IV (Complications of treatment). The secondary outcomes were: (1) Parkinson's Disease Quality of Life Questionnaire (PDQL) and Parkinson's Disease Questionnaire-39 (PDQ-39); (2) Non-motor Symptoms Questionnaire (NMSQuest) and Non-Motor Symptoms Scale (NMSS); (3) Parkinson's Disease Sleep Scale (PDSS); (4) Hamilton depression rating scale (HAMD); (5) Adverse events.

#### Exclusion criteria

Studies were excluded if they were any one of the followings: (1) clinical trials evaluating the other alternative and complementary medicines mixed in the experimental group or control group in the treatment of PD; (2) single herb, herbal extracts and herbal components; (3) case series, reviews, comments and protocols; (4) animal studies and *in vitro* studies; (5) duplicated publications.

### Quality assessment

The methodological quality was evaluated by using the Cochrane Collaboration's risk of bias tool (Higgins et al., 2011). The quality of each study was assessed by following seven biases: adequate sequence generation, allocation concealment, blinding of participants and personnel, blinding of outcome assessors, incomplete outcome data addressed (ITT analysis), free of selective reporting and other bias. Each domain can be rated as “+” (low risk of bias), “-” (high risk of bias), or “?” (unclear risk of bias), which were the three categories for the degree of each potential bias.

### Data extraction

Two authors (CS-S and HF-Z) independently extracted the data according to predefined extraction form as follows: (1) General information: the first author's name, publication year, and publication language; (2) Participants: diagnostic criteria, study design, total number and number in comparison groups, gender and mean age; (3) Intervention: herbal preparations, dose, frequency, course of treatment, follow-up; (4) Outcome measures. If the study had multiple comparison groups, we chosen the most relevant groups for analysis. The original authors were contacted if further information was needed. Disagreements were resolved through discussing with corresponding author (GZ).

The constituent of HM formulas for PD in each included study was recorded. The herbs with cumulative frequencies over 50% are documented and ranked.

### Data synthesis and statistical analysis

We synthesized all data and performed meta-analyses on RevMan 5.3 software. Continuous outcomes were using weighted mean differences (WMD) or standardized mean differences (SMD) with 95% confidence intervals (CIs), while dichotomous outcomes were summarized using risk ratio (RR) with 95% confidence intervals (CIs). Heterogeneity among studies was detected by I^2^ and Chi^2^ tests. If substantial statistical heterogeneity existed (*I*^2^ ≥ 50%, *P* < 0.10), a random-effects model was used. If there was no observed heterogeneity (*I*^2^ < 50%, *P* > 0.10), a fixed-effect model was applied. Possible sources of heterogeneity were explored by subsequent sensitivity analyses. If more than ten trials were identified in each outcome, publication bias was detected by funnel plot analyses and Egger's test.

## Results

### Description of the screening process

**Figure 1:**
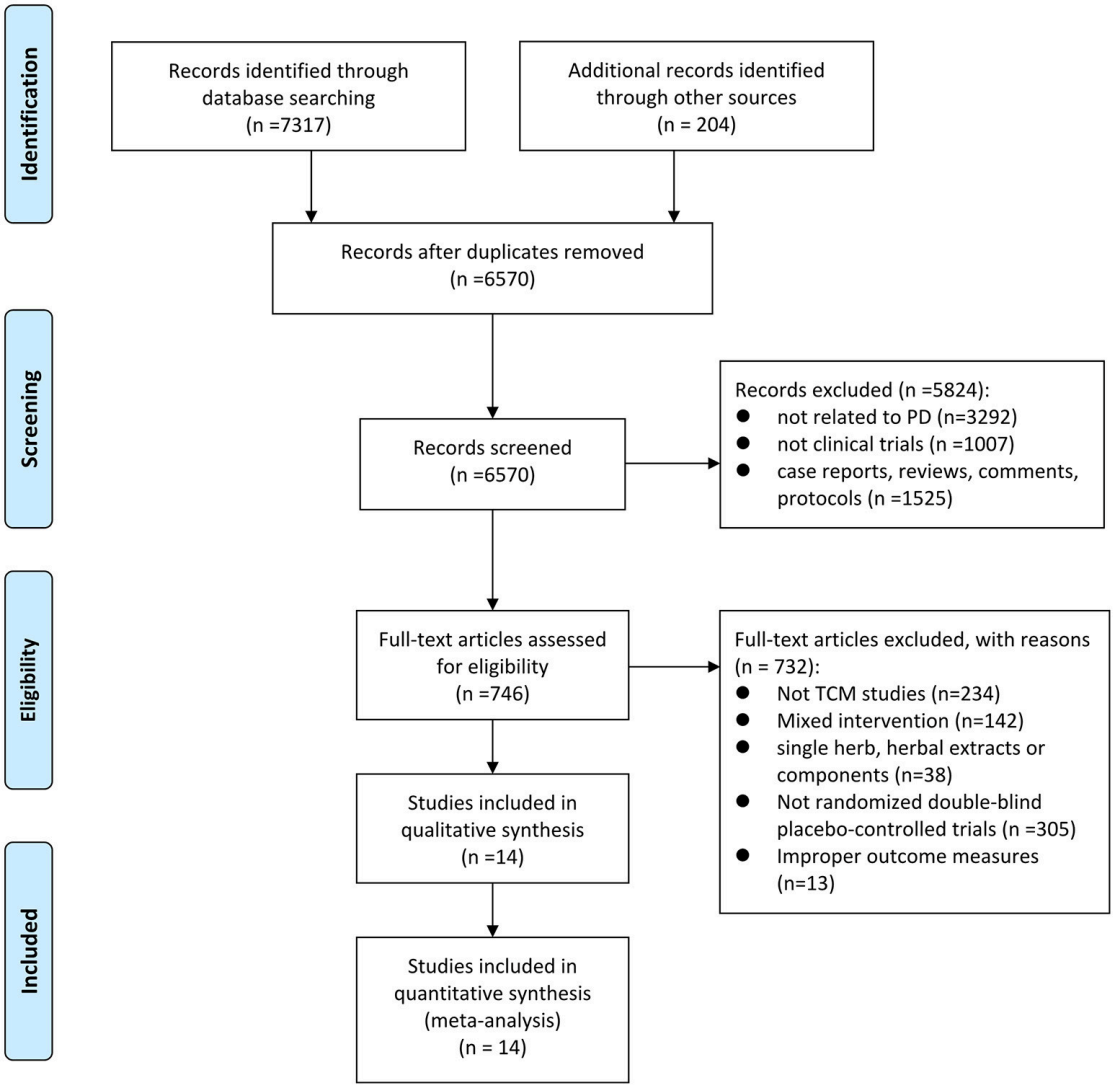
Flow diagram of the search process.

The detailed screening process was summarized in the PRISMA flow diagram (Figure 1). A total of 7,521 potentially relevant hits were initially yielded from the eight databases and other sources, in which 6,570 records were remained after removal of duplicates. Through screening titles and abstracts, we excluded 5,824 papers because they were studies with no relevance to PD (*n* = 3292), nonclinical trials (*n* = 1007), case reports, reviews, comments OR protocols (*n* = 1525). After full-text evaluation, 732 papers were excluded, including 234 that were not CHM studies, 142 that contained mixed interventions, 38 that aimed at single herb, herbal extracts or components, 305 that were not randomized double-blind placebo-controlled trials, and 13 that observed no outcome of interest. Ultimately, 14 eligible studies (Pan et al., 2009, 2011, 2013; Zhao et al., 2009, 2013; Guo, 2010; Kum et al., 2011; Chen M. Y. et al., 2014; Guo et al., 2014; Wen et al., 2015; Li et al., 2016; Yu, 2016; Cai et al., 2017; Yang, 2017) were selected in our study.

## Study characteristics

The general characteristics of the included studies are summarized in Table 1. Fourteen included studies (Pan et al., 2009, 2011, 2013; Zhao et al., 2009, 2013; Guo, 2010; Kum et al., 2011; Chen M. Y. et al., 2014; Guo et al., 2014; Wen et al., 2015; Li et al., 2016; Yu, 2016; Cai et al., 2017; Yang, 2017) were published between 2009 and 2017. Among them, 4 studies (Kum et al., 2011; Pan et al., 2011, 2013; Li et al., 2016) were published in English and 10 studies (Pan et al., 2009; Zhao et al., 2009, 2013; Guo, 2010; Chen M. Y. et al., 2014; Guo et al., 2014; Wen et al., 2015; Yu, 2016; Cai et al., 2017; Yang, 2017) in Chinese. The most used diagnostic criterion of PD was UK Brain Bank criteria, which was referred in 11 studies (Guo, 2010; Kum et al., 2011; Pan et al., 2011, 2013; Zhao et al., 2013; Chen M. Y. et al., 2014; Guo et al., 2014; Wen et al., 2015; Li et al., 2016; Cai et al., 2017; Yang, 2017). Comparison of HM monotherapy vs. placebo was performed in one trial (Zhao et al., 2009). Comparisons of CHM plus WCM versus placebo plus WCM were conducted in 14 trials (Pan et al., 2009, 2011, 2013; Zhao et al., 2009, 2013; Guo, 2010; Kum et al., 2011; Chen M. Y. et al., 2014; Guo et al., 2014; Wen et al., 2015; Li et al., 2016; Yu, 2016; Cai et al., 2017; Yang, 2017), of whom 7 trials used Madopar (Zhao et al., 2009; Guo, 2010; Guo et al., 2014; Li et al., 2016; Yu, 2016; Cai et al., 2017; Yang, 2017). All studies involved a total of 1,311 patients with 675 in the treatment group vs. 636 in the placebo group, ranging in age from 51 to 79 years old. The sample size of the included studies ranged from 47 to 242. The male-to-female ratio was between 1.0 and 2.1. Duration of disease ranged from 2.2 months to 11.3 years. The total intervention period varied from 8 weeks to 6 months. The most common duration was 12 weeks. Two studies (Pan et al., 2013; Li et al., 2016) mentioned the follow-up times were 4 weeks and 6 months, respectively.

**Table 1:** Characteristics of the included studies.

**Included studies**	**Publication language**	**Diagnostic criteria**	**Study designs**	**No. of participants (male/female); mean age (years)**		**Course of disease**		**Interventions**		**Course of treatment**	**Follow up**	**Outcome index**	**Intergroup differences**
				**Trial**	**Control**	**Trial**	**Control**	**Trial**	**Control**			
Cai et al., 2017	Chinese	UK brain bank standard	Randomized double-blind and placebo-controlled parallel study	43 (25/18) 57.24 ± 3.36	43 (23/20) 58.14 ± 4.12	4.45 ± 1.36 years	4.75 ± 1.68 years	1. Zhichan decoction (1 package, bid)2. Madopar and Sinemet (NR)	1.Placebo (1 package, bid) 2. Madopar and Sinemet (NR)	12 weeks	NR	1. UPDRS I 2. UPDRS II 3. UPDRS III 4. UPDRS IV 5. Total UPDRS Score 6. PDQL 7. NMSQuest 8. PDSS 9. HAMD 10. Adverse events	1. *P* < 0.05 2. *P* < 0.05 3. *P* < 0.05 4.*P* > 0.05 5. *P* < 0.05 6. *P* < 0.01 7. *P* < 0.01 8. *P* < 0.01 9. *P* < 0.01 10. *P* < 0.05
Chen M. Y. et al., 2014	Chinese	UK brain bank standard	Randomized, double-blind and placebo-controlled parallel study	57 (38/19) 66.44 ± 7.64	51 (35/16) 65.63 ± 7.37	4.41 ± 2.45 years	4.39 ± 3.07 years	1.Zhichan granule (1 package, bid)2.WCM (NR)	1.Placebo (1 package, bid) 2. WCM (NR)	12 weeks	NR	1. UPDRS I2. UPDRS II 3. UPDRS III 4. UPDRS IV 5. Total UPDRS Score 6. Adverse events	1.*P* > 0.05 2.*P* > 0.05 3.*P* < 0.05 4.*P* > 0.05 5. *P* < 0.05 6.*P* > 0.05
Guo, 2010	Chinese	UK brain bank standard	Randomized, double-blind and placebo-controlled parallel study	30 (20/10) 68.23 ± 7.22	30 (16/14) 67.47 ± 8.12	47.4 ± 33.98 months	39.67 ± 24.33 months	1.Guilu Dihuang capsule (1.5 g, tid)2. Madopar (125 mg, tid)	1.Placebo (1.5 g, tid) 2. Madopar (125 mg, tid)	8 weeks	NR	1. Adverse events	1. *P* > 0.05
Guo et al., 2014	Chinese	UK brain bank standard	Randomized, double-blind and placebo-controlled parallel study	35 (NR)NR	30 (NR) NR	NR	NR	1. Bushen Huoxue granules (1 package, bid) 2. Madopar (NR)	1. Placebo (1 package, bid) 2. Madopar (NR)	6 months	NR	1. HAMD 2. Adverse Events	1. *P* > 0.01 2. *P* > 0.05
Kum et al., 2011	English	UK brain bank standard	Randomized, double-blind and placebo-controlled parallel study	22 (14/8) 64.82 ± 8.88	25 (17/8) 60.88 ± 9.41	5.44 ± 5.26 years	6.37 ± 4.93 years	1.Jiawei Liujunzi Tang granule (1 package, qd) 2.Levodopa (NR)	1.Placebo (1 package, qd) 2.Levodopa (NR)	24 weeks	NR	1. UPDRS IV 2. PDQ-39 3. Adverse Events	1. *P* < 0.05 2. *P* < 0.05 3. *P* > 0.05
Li et al., 2016	English	UK brain bank Standard	Multi-center randomized, double-blind and placebo-controlled parallel study	60 (31/27) 66.6 ± 1.2	60 (42/20) 67.3 ± 1.2	5.2 ± 0.4 years	5.1 ± 0.5 years	1.Bushen Huoxue granule (1 package, bid) 2. Madopar (NR)	1.Placebo (1 package, bid) 2. Madopar (NR)	3 months	6 months	1. UPDRS II2. UPDRS III3. PDQ-394. PDSS5. Adverse Events	1. *P* > 0.05 2. *P* > 0.05 3. *P* > 0.05 4. *P* > 0.05 5. *P* > 0.05
Pan et al., 2009	Chinese	JDS for PD in 1997	Randomized, double-blind and placebo-controlled parallel study	32 (18/14) 64.9 ± 10.2	18 (10/8) 65.3 ± 10.2	4.9 ± 4 years	5.1 ± 2.5 years	1.Zengxiao Anshen Zhichan 2 granule (5 g, bid) 2. WCM (NR)	1.Placebo (5 g, bid) 2. WCM (NR)	3 months	NR	1. UPDRS I2. UPDRS II 3. UPDRS III 4. UPDRS IV 5. Total UPDRS Score	1. *P* > 0.05 2. *P* > 0.05 3. *P* > 0.05 4. *P* > 0.05 5. *P* > 0.05
				30 (16/14) 65.1 ± 9.4	16 (8/8) 63.7 ± 11.1	6.1 ± 3.8 years	6.3 ± 2.9 years	1.Zengxiao Anshen Zhichan 2 granule (5 g, bid)2. WCM (NR)	1.Placebo (5 g, bid) 2. WCM (NR)	3 months	NR	1. UPDRS I 2. UPDRS II 3. UPDRS III 4. UPDRS IV 5. Total UPDRS Score	1. *P* > 0.05 2. *P* > 0.05 3. *P* > 0.05 4. *P* > 0.05 5. *P* > 0.05
Pan et al., 2011	English	UK brain bank standard	Randomized, double-blind and placebo-controlled parallel study	56 (34/22) 62.82 ± 10.31	54 (32/22) 63.1 ± 10.2	5.73 ± 4.81 years	5.81 ± 3.24 years	1.Zengxiao Anshen Zhichan 2 granule (1 package, tid)2. WCM (NR)	1. Placebo (1 package, tid) 2. WCM (NR)	3 months	NR	1. UPDRS I2. UPDRS II 3. UPDRS III 4. UPDRS IV 5. Total UPDRS Score 6. Adverse Events	1. *P* > 0.05 2. *P* < 0.05 3. *P* > 0.05 4. *P* < 0.05 5. *P* < 0.05 6. *P* > 0.05
Pan et al., 2013	English	UK brain bank standard	Randomized, double-blind and placebo-controlled parallel study	31 (18/13) 68.6 ± 9.2	30 (19/11) 67.1 ± 10.2	5.9 ± 4.7 years	6.1 ± 4.9 years	1.Yangxue Qingnao granule (1 package, tid)2. WCM (NR)	1.Placebo (1 package, tid) 2. WCM (NR)	12 weeks	4 weeks	1. PDSS 2. Adverse events	1. *P* < 0.05 2. *P* > 0.05
Wen et al., 2015	Chinese	UK brain bank standard	Randomized, double-blind and placebo-controlled parallel study	29 (15/14) 70.79 ± 7.5	28 (16/12) 71.21 ± 5.96	34.48 ± 12.70 m	34.78 ± 11.63 m	1.Congwu Qufeng granule (1 package, tid)2. WCM (NR)	1.Placebo (1 package, tid) 2. WCM (NR)	3 months	NR	1. PDQ-39	1. *P* < 0.05
Yang, 2017	Chinese	UK brain bank standard	Randomized, double-blind and placebo-controlled parallel study	40 (19/21) 65.24 ± 12.53	39 (21/18) 63.28 ± 11.26	7.02 ± 3.63 years	6.24 ± 2.16 years	1.Yishen Chuchan decoction (0.5 dose, bid) 2. Madopar (NR)	1.Placebo (0.5 dose, bid) 2. Madopar (NR)	2 months	NR	1. UPDRS III2. PDQ-393. NMSS4. Adverse Events	1.*P* < 0.05 2. *P* < 0.01 3. *P* < 0.01 4. *P* > 0.05
Yu, 2016	Chinese	CNDS for PD in 2006	Randomized, double-blind and placebo-controlled parallel study	34 (19/15) 70.765 ± 7.836	34 (17/17) 69.706 ± 9.137	24.176 ± 10.715 months	26.029 ± 11.371 months	1.Naokang granule (1 package, tid)2. Madopar (125 mg, tid)	1.Placebo (1 package, bid) 2. Madopar (125 mg, tid)	2 months	NR	1. UPDRS II2. UPDRS III3. NMSS4. Adverse Events	1. *P* < 0.05 2. *P* < 0.05 3. *P* < 0.05 4. *P* > 0.05
Zhao et al., 2009	Chinese	CNDS for PD in 1984	Multi-center randomized, double-blind and placebo-controlled parallel study	28 (15/13) 65.40 ± 8.16	25 (15/10) 63.14 ± 11.58	3.69 ± 1.82 years	4.19 ± 3.39 years	Guiling Pa'an capsule (1.5 g, tid)	Placebo (1.5 g, tid)	12 weeks	NR	1. UPDRS II2. UPDRS III3. Total UPDRS Score	1. *P* > 0.05 2. *P* > 0.05 3. *P* > 0.05
				75 (46/29) 64.86 ± 9.85	79 (47/32) 65.63 ± 8.51	4.7 ± 3.44 years	4.59 ± 3.82 years	1.Guiling Pa'an capsule (3 g, tid)2. Madopar and Sinemet (NR)	1.Placebo (3 g, tid) 2. Madopar and Sinemet (NR)	12 weeks	NR	1. UPDRS II2. UPDRS III3. Total UPDRS Score	1. *P* > 0.05 2. *P* > 0.05 3. *P* > 0.05
				19 (8/11) 67.24 ± 9.54	16 (12/4) 66.l 0 ± 7.61	6.24 ± 4.31 years	6.26 ± 2.53 years	1.Guiling Pa'an capsule (3 g, tid)2. Madopar and Sinemet (NR)	1, Placebo (3 g, tid) 2. Madopar and Sinemet (NR)	12 weeks	NR	1. UPDRS II2. UPDRS III3. Total UPDRS Score	1. *P* > 0.05 2. *P* > 0.05 3. *P* > 0.05
Zhao et al., 2013	Chinese	UK brain bank standard	Multicenter randomized, double-blind and placebo-controlled parallel study	54 (42/16) 68.64 ± 8.00	58 (27/27) 68.46 ± 8.80	2–18 years	2–21 years	1.Guiling Pa'an granule (6 g, tid)2. WCM (NR)	1.Placebo (6 g, tid) 2. WCM (NR)	6 months	NR	1. Adverse Events	1. *P* > 0.05

*bid, bis in die; CNDS, Chinese National Diagnosis Standard; HAMD, Hamilton depression rating scale; JDS, Japnese Diagnosis Standard; m, month; NMSQuest, Non-motor Symptoms Questionnaire; NMSS, Non-Motor Symptoms Scale; NR, not reported; PDQ-39, Parkinson's Disease Questionnaire-39; PDQL, Parkinson's Disease Quality of Life Questionnaire; PDSS, Parkinson's Disease Sleep Scale; qd, quaque die; tid, ter in die; UPDRS, Unified Parkinson's Disease Rating Scale; w, week; WCM, western conventional medication; y, year*.

### Description of the HM formulas

Fourteen studies reported a wide range of TCM formulas, including Bushen Huoxue granule (*n* = 2), Zengxiao Anshen Zhichan 2 capsule (*n* = 1), Zengxiao Anshen Zhichan 2 granule (*n* = 1), Zhichan decoction (*n* = 1), Zhichan granule (*n* = 1), Guilu Dihuang capsule (*n* = 1), Jiawei Liujunzi Tang granule (*n* = 1), Yangxue Qingnao granule (*n* = 1), Congwu Qufeng granule (*n* = 1), Yishen Chuchan decoction (*n* = 1), Naokang granule (*n* = 1), Guiling Pa'an capsule (*n* = 1), and Guiling Pa'an granule (*n* = 1). The ingredients of TCM formulas in each included studies were presented in Table 2. A total of 52 herbs were used in these TCM formulas. High-frequency herbs in HM formulas were ranked in Table 3. The top 11 most frequently used herbs were Radix Salviae Miltiorrhizae (Dan Shen), Radix Paeoniae Alba (Bai Shao), Ramulus Uncariae Cum Uncis (Gou Teng), Radix Rehmanniae (Di Huang), Herba Cistanches (Rou Cong Rong), Radix Polygoni Multiflori (He Shou Wu), Rhizoma Ligustici Chuanxiong (Chuan Xiong), Fructus Corni (Shan Zhu Yu), Radix Angelicae Sinensis (Dang Gui), Rhizoma Acori Tatarinowii (Shi Chang Pu), and Radix Astragali seu Hedysari (Huang Qi).

**Table 2:** Ingredients of TCM formula.

**Included studies**	**Prescription**	**Ingredients**		
		**Latin name**	**English name**	**Chinese name**
Cai et al., 2017	Zhichan decoction	Radix Astragali seu Hedysari, Radix Paeoniae Alba, Radix Salviae Miltiorrhizae, Rhizoma Anemarrhenae, Ramulus Uncariae Cum Uncis, Rhizoma Cimicifugae, Rhizoma Polygoni Cuspidati.	Milkvetch root, debark peony root, danshen root, common anemarrhena rhizome, gambir plant nod, largetrifoliolious bugbane rhizome, giant knotweed rhizome.	Huang Qi, Bai Shao, Dan Shen, Zhi Mu, Gou Teng, Sheng Ma, Hu Zhang.
Chen M. Y. et al., 2014	Zhichan granule	Radix Astragali seu Hedysari, Ramulus Uncariae Cum Uncis, Radix Polygoni Multiflori Preparata, Radix Paeoniae Alba, Rhizoma Anemarrhenae, et al.	Milkvetch root, gambir plant nod, prepared fleeceflower root, debark peony root, common anemarrhena rhizome, et al.	Huang Qi, Gou Teng, Zhi He Shou Wu, Bai Shao, Zhi Mu, et al.
Guo, 2010	Guilu Dihuang capsule	Radix Rehmanniae Preparata, Chinemys reevesii, Colla Corni Cervi, et al.	Prepared rehmannia root, tortoise plastron glue, deerhorn glue, et al.	Shu Di Huang, Gui Ban Jiao, Lu Jiao Jiao, et al.
Guo et al., 2014	Bushen Huoxue granule	Fructus Corni, Herba Cistanches, Radix Polygoni Multiflori, Rhizoma Ligustici Chuanxiong, Radix Angelicae Sinensis, Radix Salviae Miltiorrhizae, Scolopendra, et al.	Asiatic cornelian cherry fruit, desertliving cistanche, fleeceflower root, sichuan lovage rhizome, Chinese angelica, danshen root, centipede, et al.	Shan Zhu Yu; Rou Cong Rong; He Shou Wu; Chuan Xiong; Dang Gui; Dan Shen; Wu Gong , et al.
Kum et al., 2011	Jiawei Liujun Zi Tang granule	Radix Codonopsis, Radix Rehmanniae Recens, Poria, Ramulus Uncariae Cum Uncis, Rhizoma Atractylodis Macrocephalae, Radix Angelicae Sinensis, Rhizoma Pinelliae Preparatum, Rhizoma Ligustici Chuanxiong, Radix Achyranthis Bidentatae, Pericarpium Citri Reticulatae, Radix Glycyrrhizae.	Tangshen, unprocessed rehmannia root, Indian bread, gambir plant nod, largehead atractylodes rhizome, Chinese angelica, processed pinellia tuber, sichuan lovage rhizome, twotoothed achyranthes root, dried tangerine peel, liquorice root.	Dang shen; Sheng di huang; Fu ling; Gou Teng; Bai Zhu; Dang Gui; Fa ban xia; Chuan Xiong; niu xi; Chen pi; Gan cao.
Li et al., 2016	Bushen Huoxue granule	Fructus Corni, Rhizoma Acor tatarinowii, Radix Polygoni multiflori, Herba Cistanches, Raix Angelicae sinensis, Radix Salviae miltiorrhizae, Scolopendra.	Asiatic cornelian cherry fruit, grassleaf sweetflag rhizome, fleeceflower root, desertliving cistanche, Chinese angelica, danshen root, centipede.	Shan Zhu Yu, Shi Chang Pu, He Shou Wu; Rou Cong Rong, Dang Gui; Dan Shen; Wu Gong.
Pan et al., 2009	Zeng-xiao An-shen Zhi-chan 2 capsule	Radix Rehmanniae Preparata, Fructus Corni, Os Draconis, Radix Asparagi, Radix Paeoniae Alba, Carapax et Plastrum Testudinis, Herba Cistanches, Radix Puerariae, Rhizoma Arisaematis Cum Bile, Scorpio, Radix Salviae Miltiorrhizae, Lumbricus, Rhizoma Acori Tatarinowii, Rhizoma Curcumae Longae.	Prepared rehmannia root, asiatic cornelian cherry fruit, bone fossil of big mammals, cochinchinese asparagus root, debark peony root, tortoise carapace and plastron, desertliving cistanche, kudzuvine root, bile arisaema, scorpion, Danshen root, earthworm, grassleaf sweetflag rhizome, turmeric.	Shu Di Huang; Shan Zhu Yu; Long Gu; Tian Dong; Shao Yao; Gui Jia; Rou Cong Rong; Ge Gen; Dan Nan Xing; Quan Xie; Dan Shen; Di Long; Shi Chang Pu; Jiang Huang.
Pan et al., 2011	Zengxiao Anshen Zhichan 2 granule	Ramulus Uncariae Cum Uncis, Radix Rehmanniae Recens, Fructus Corni, Radix Asparagi, Radix Paeoniae Alba, Herba Cistanches, Radix Puerariae, Rhizoma Arisaematis, Radix Salviae Miltiorrhizae, Rhizoma Acori Tatarinowii, Rhizoma Curcumae Longae, Radix Morindae Officinalis, Rhizoma Gastrodiae, Rhizoma Ligustici Chuanxiong,	Gambir plant nod, unprocessed rehmannia root, asiatic cornelian cherry fruit, cochinchinese asparagus root, debark peony root, desertliving cistanche, kudzuvine root, jackinthepulpit tuber, danshen root, grassleaf sweetflag rhizome, turmeric, morinda root, tall gastrodia tuber, sichuan lovage rhizome.	Gou Teng; Sheng Di Huang; Shan Zhu Yu; Tian Dong; Bai Shao; Rou Cong Rong; Ge Gen; Tian Nan Xing; Dan Shen; Shi Chang Pu; Jiang Huang; Ba Ji Tian; Tian Ma; Chuan Xiong.
Pan et al., 2013	Yangxue Qingnao granule	Radix Angelicae Sinensis, Rhizoma Ligustici Chuanxiong, Radix Paeoniae Alba, Ramulus Uncariae Cum Uncis, Caulis Spatholobi, Spica Prunellae, Concha Margaritifera, Radix Rehmanniae Recens, Semen Cassiae, Rhizoma Corydalis, Herba Asari.	Chinese angelica, sichuan lovage Rhizome, debark peony root, gambir plant nod, suberect spatholobus stem, common selfheal fruit-spike, nacre, unprocessed rehmannia root, cassia seed, yanhusuo, manchurian wildginger.	Dang Gui; Chuan Xiong; Bai shao; Gou Teng; Ji Xue Teng; Xia Ku Cao; Zhen Zhu Mu; Di Huang; Jue Ming Zi; Yan Hu Suo; Xi Xin
Wen et al., 2015	Congwu Xifeng granule	Radix Polygoni Multiflori Preparata, Herba Cistanches, Cortex Eucommiae, Rhizoma Gastrodiae Ramulus Uncariae Cum Uncis, Rhizoma Atractylodis Macrocephalae, Radix Paeoniae Alba, Rhizoma Polygoni Cuspidati, Radix et Rhizoma Rhei,	Prepared fleeceflower root, desertliving cistanche, eucommia bark, tall gastrodia tuber, gambir plant nod, largehead atractylodes rhizome, debark peony root, giant knotweed rhizome, rhubarb root and rhizome.	Zhi He Shou Wu; Rou Cong Rong; Du Zhong; Tian Ma , Gou Teng; Bai Zhu; Bai Shao; Hu Zhang; Jiu Da Huang.
Yang et al., 2017	Yishen Chuchan decoction	Radix Polygoni Multiflori Preparata, Radix Rehmanniae Recens, Rhizoma Gastrodiae, Radix Paeoniae Alba, Concha Ostreae, Bombyx Batryticatus, Radix et Rhizoma Rhei, Radix Linderae, Rhizoma Dioscoreae, Fructus Alpiniae oxyphyllae.	PREPARED fleeceflower root, unprocessed rehmannia root, tall gastrodia tuber, debark peony root, oyster shell, stiff silkworm, rhubarb root and rhizome, combined spicebush root, common yam rhizome, sharp-leaf glangal fruit.	Zhi He Shou Wu, Sheng Di Huang, Tian Ma, Bai Shao, Mu Li, Jiang Can, Da Huang, wu Yao, Shan yao, Yi Zhi.
Yu, 2016	Naokang granule	Herba Cistanches, Radix Notoginseng, Rhizoma Ligustici Chuanxiong, Radix Salviae Miltiorrhizae, Rhizoma Acori Tatarinowii, Radix Polygalae, Scolopendra, Lumbricus, Bombyx Batryticatus, Scorpio, Radix Astragali seu Hedysari, Radix Codonopsis, Herba Epimedii.	Desertliving cistanche, sanqi, sichuan lovage rhizome, danshen root, grassleaf sweetflag rhizome, milkwort root, centipede, earthworm, stiff silkworm, scorpion, milkvetch root, tangshen, epimedium herb.	Rou Cong Rong, San Qi, Chuan Xiong, Dan Shen, Shi Chang Pu, Yuan Zhi, Wu Gong, Di Long, Jiang Can, Quan Xie, Huang qi, Dang Shen, Yin Yang Huo.
Zhao et al., 2009, 2013	Guiling Pa'an capsule Guiling Pa'an granule	Carapax et Plastrum Testudinis, Cornu Saigae Tataricae, et al.	Tortoise carapace and plastron, antelope horn, et al.	Gui Jia; Ling Yang Jiao, et al.

**Table 3:** The 11 high-frequency used herbs for PD in the 14 trials included.

**Herb name**			**Frequency**	**The total frequency %**	**Cumulative percentiles %**
**Latin name**	**English name**	**Chinese name**		
Radix Salviae Miltiorrhizae	Danshen root	Dan Shen	7	6.1	6.1
Radix Paeoniae Alba	Debark peony root	Bai Shao	7	6.1	12.2
Ramulus Uncariae Cum Uncis	Gambir plant nod	Gou Teng	6	5.2	17.4
Radix Rehmanniae	Rehmannia root	Di Huang	6	5.2	22.6
Herba Cistanches	Desertliving cistanche	Rou Cong Rong	6	5.2	27.8
Radix Polygoni Multiflori	Fleeceflower root	He Shou Wu	5	4.3	32.2
Rhizoma Ligustici Chuanxiong	Sichuan lovage rhizome	Chuan Xiong	5	4.3	36.5
Fructus Corni	Asiatic cornelian cherry fruit	Shan Zhu Yu	4	3.5	40.0
Radix Angelicae Sinensis	Chinese angelica	Dang Gui	4	3.5	43.5
Rhizoma Acori Tatarinowii	Grassleaf sweetflag rhizome	Shi Chang Pu	4	3.5	47.0
Radix Astragali seu Hedysari	Milkvetch root	Huang Qi	3	2.6	49.6

### Assessing the quality of studies

**Figure 2:**
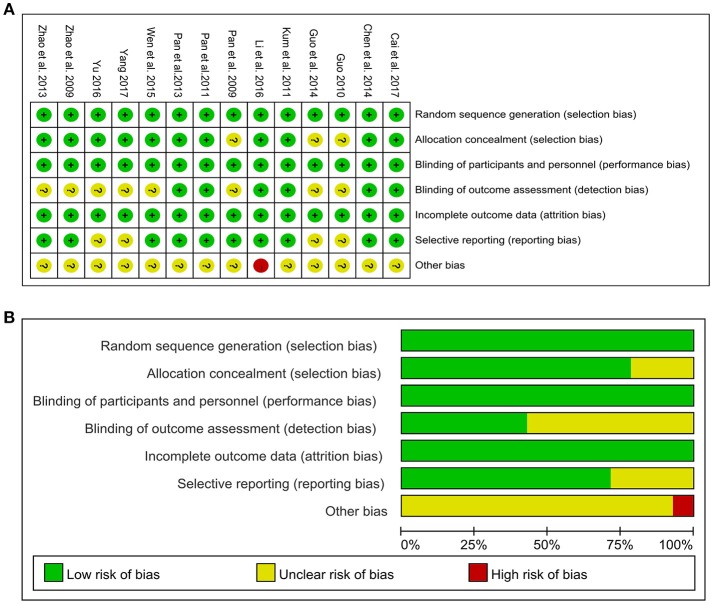
Risk of bias of the included studies. **(A)** Risk of bias summary: judgements about each risk of bias item for each included study. **(B)** Risk of bias graph: judgements about each risk of bias item presented as percentages across all included studies. +, low risk of bias; -, high risk of bias; ?, unclear risk of bias.

The methodological quality of all included studies was detailed in the Figure 2. All included studies were randomized studies with explicit description. Specifically, seven studies (Pan et al., 2009, 2011; Guo et al., 2014; Wen et al., 2015; Yu, 2016; Cai et al., 2017; Yang, 2017) used random number tables. Four studies (Zhao et al., 2009; Kum et al., 2011; Pan et al., 2013; Chen M. Y. et al., 2014) used computer-generated lists of random numbers. Two studies (Zhao et al., 2013; Li et al., 2016) employed online center distribution, while only one study (Guo, 2010) stated the method for sequence generation by simple randomization. Eleven studies (Zhao et al., 2009, 2013; Kum et al., 2011; Pan et al., 2011, 2013; Chen M. Y. et al., 2014; Wen et al., 2015; Li et al., 2016; Yu, 2016; Cai et al., 2017; Yang, 2017) reported adequate allocation concealment. Four (Kum et al., 2011; Chen M. Y. et al., 2014; Yu, 2016; Cai et al., 2017) adopted opaque and sealed envelopes. The remaining studies (Zhao et al., 2009, 2013; Pan et al., 2011, 2013; Wen et al., 2015; Li et al., 2016; Yang, 2017) adopted center distribution. Of 14 included studies, 8 studies (Pan et al., 2009; Zhao et al., 2009, 2013; Guo, 2010; Guo et al., 2014; Wen et al., 2015; Yu, 2016; Yang, 2017) applied double blinding and 6 studies (Kum et al., 2011; Pan et al., 2011, 2013; Chen M. Y. et al., 2014; Li et al., 2016; Cai et al., 2017) had triple blinding. All studies had low risk of bias in the incomplete out-come data. Four studies (Guo, 2010; Guo et al., 2014; Yu, 2016; Yang, 2017) had unclear risk of bias in selective reporting because of no available protocols. Other risks of bias were described in one study (Li et al., 2016), which reported significant differences in baseline values of some outcome variables.

### Effect estimation

#### HM monotherapy vs. placebo

One study (Zhao et al., 2009) showed that the efficacy of HM monotherapy was similar to placebo according to UPDRS II (*P* > 0.05), UPDRS III (*P* > 0.05) and total UPDRS score (*P* > 0.05) in PD patients who never received levodopa treatment.

#### HM plus WCM vs. placebo plus WCM

**Figure 3:**
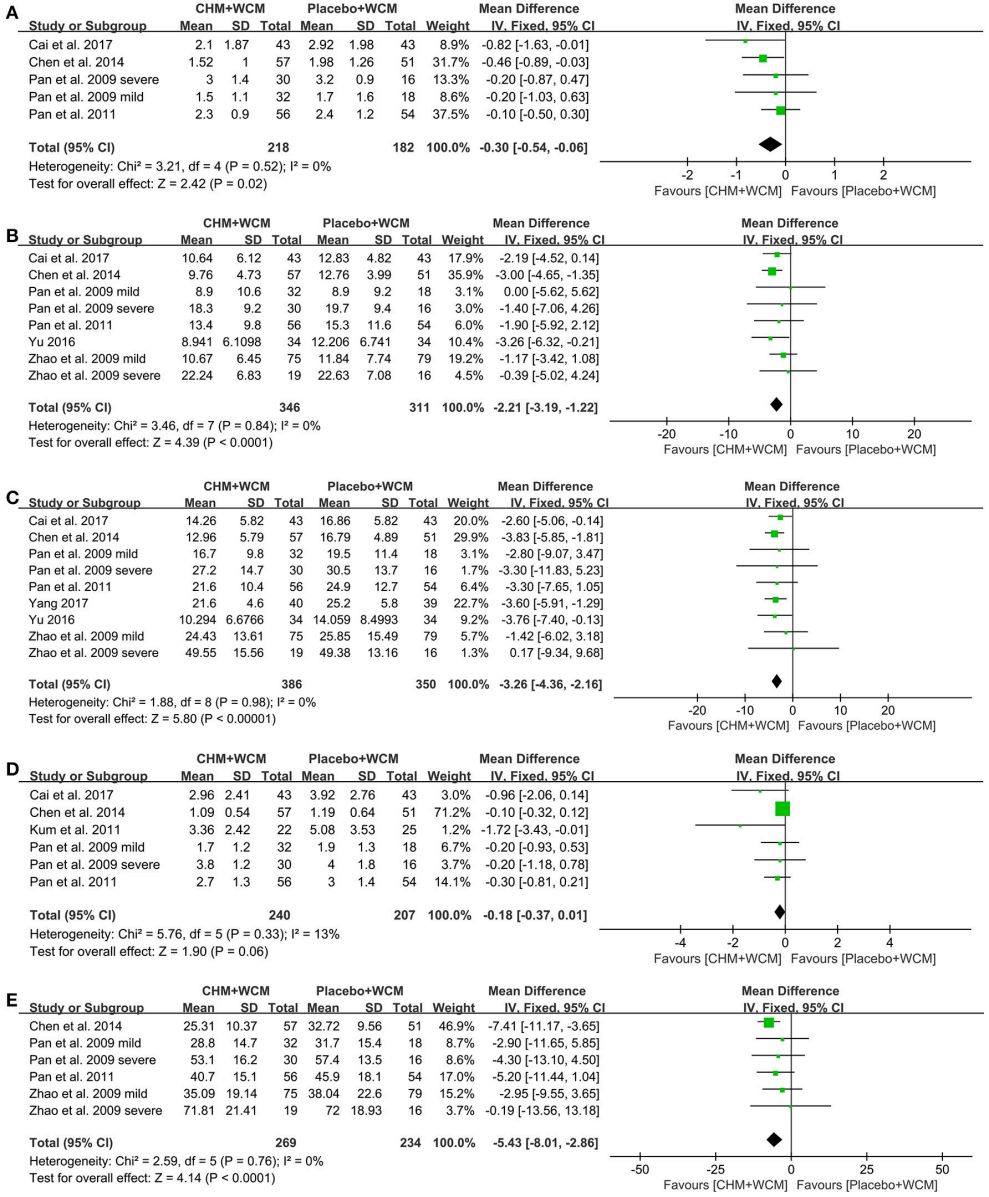
Forest plot of HM plus WCM vs. placebo plus WCM in terms of **(A)** UPDRS I, **(B)** UPDRS II, **(C)** UPDRS III, **(D)** UPDRS IV, and **(E)** Total UPDRS Score. (HM, herbal medicine; WCM, western conventional medicine; UPDRS, Unified Parkinson's Disease Rating Scale).

**UPDRS I:** Four studies (Pan et al., 2009, 2011; Chen M. Y. et al., 2014; Cai et al., 2017) with 5 comparisons showed that the HM paratherapy significantly improved UPDRS I compared with control groups (WMD: −0.30, 95% CI: −0.54 to −0.06, *P* = 0.02; heterogeneity: Chi^2^ = 3.21, *P* = 0.52, *I*^2^ = 0%; Figure 3A).

**UPDRS II:** Seven studies (Pan et al., 2009, 2011; Zhao et al., 2009; Chen M. Y. et al., 2014; Li et al., 2016; Yu, 2016; Cai et al., 2017) with 9 comparisons assessed UPDRS II. Compared with the placebo, meta-analysis of 9 comparisons showed that HM paratherapy significantly improved UPDRS II (WMD: −1.53, 95% CI −2.76 to −0.30, *P* = 0.01; heterogeneity: Chi^2^ = 19.42, *P* = 0.01, *I*^2^ = 59%). Sensitivity analyses conducted to explore potential sources of heterogeneity. A trial (Li et al., 2016) had imbalanced baseline comparing HM with placebo. After removing the trial, meta-analysis of 8 comparisons (Pan et al., 2009, 2011; Zhao et al., 2009; Chen M. Y. et al., 2014; Yu, 2016; Cai et al., 2017) showed that HM paratherapy was still superior to the placebo (WMD: −2.21, 95% CI: −3.19 to −1.22, *P* < 0.0001; heterogeneity: Chi^2^ = 3.46, *P* = 0.84, *I*^2^ = 0%; Figure 3B).

**UPDRS III:** Eight studies (Pan et al., 2009, 2011; Zhao et al., 2009; Chen M. Y. et al., 2014; Li et al., 2016; Yu, 2016; Cai et al., 2017; Yang, 2017) with 10 comparisons used UPDRS III as outcome measure. Compared with the placebo, meta-analysis of 10 comparisons showed that HM paratherapy had no significance for improving UPDRS III (WMD = −2.13, 95% CI:−4.92 to 0.66, *P* = 0.19; heterogeneity: Chi^2^ = 87.10, *P* < 0.00001, *I*^2^ = 90%). A trial (Li et al., 2016) had imbalanced baseline comparing HM with placebo. After removing the trial, meta-analysis of 9 comparisons (Pan et al., 2009, 2011; Zhao et al., 2009; Chen M. Y. et al., 2014; Yu, 2016; Cai et al., 2017; Yang, 2017) showed that HM paratherapy was superior to the placebo (WMD: −3.26, 95% CI:−4.36 to −2.16, *P* < 0.00001; heterogeneity: Chi^2^ = 1.88, *P* = 0.98, *I*^2^ = 0%; Figure 3C).

**UPDRS IV:** Meta-analysis of 5 studies (Pan et al., 2009, 2011; Kum et al., 2011; Chen M. Y. et al., 2014; Cai et al., 2017) with 6 comparisons revealed that HM paratherapy did not significantly improve UPDRS IV relative to placebo (WMD: −0.18, 95% CI: −0.37 to −0.01, *P* = 0.06; heterogeneity: Chi^2^ = 5.76, *P* = 0.33, *I*^2^ = 13%; Figure 3D).

**Total UPDRS Score:** Meta-analysis of 4 studies (Pan et al., 2009, 2011; Zhao et al., 2009; Chen M. Y. et al., 2014) with 6 comparisons revealed that HM paratherapy significantly improved the total UPDRS scores relative to placebo (WMD: −5.43, 95% CI:−8.01 to −2.86, *P* < 0.0001; heterogeneity: Chi^2^ = 2.59, *P* = 0.76, *I*^2^ = 0%; Figure 3E).

**Figure 4:**
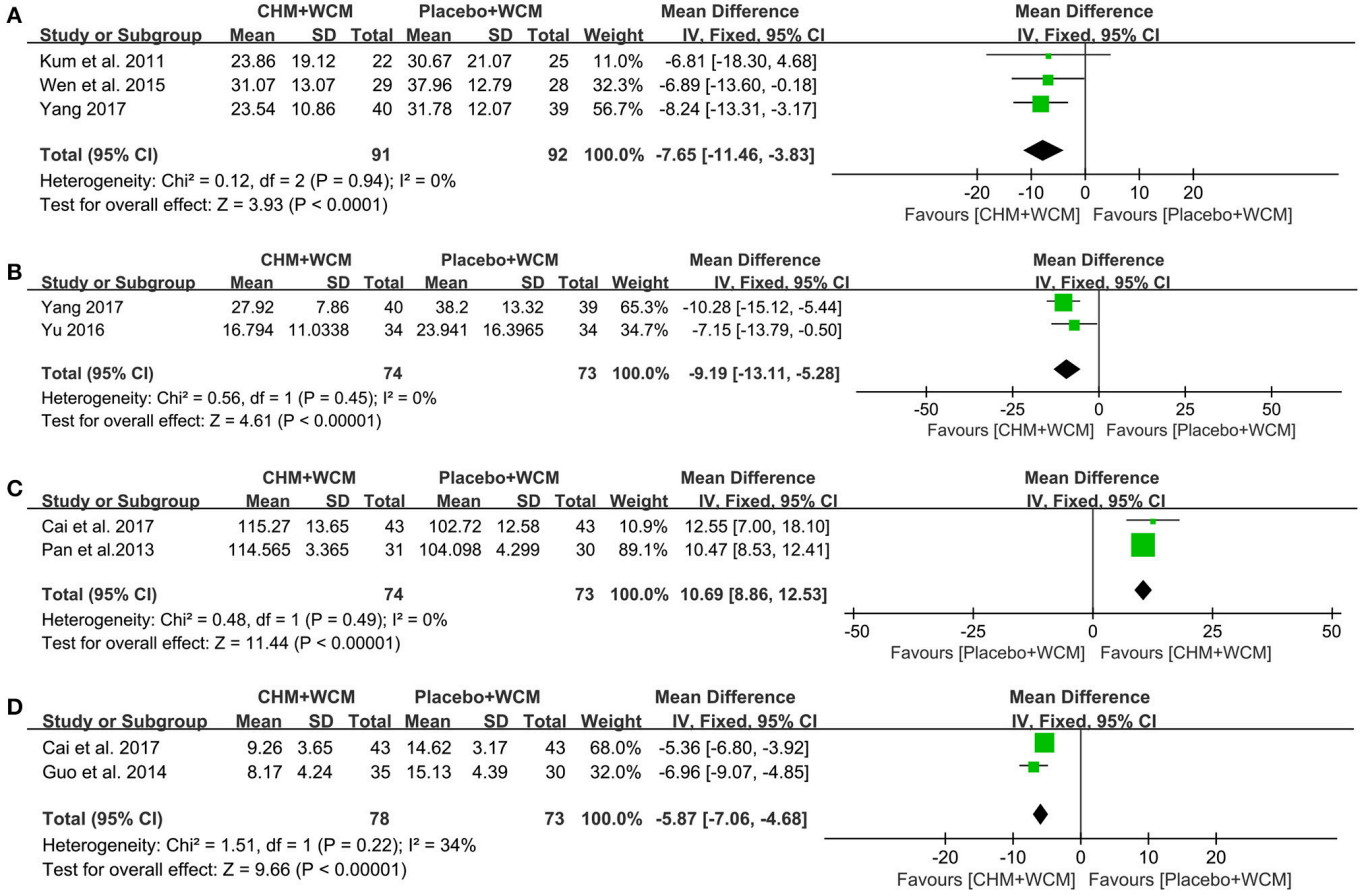
Forest plot of HM plus WCM vs. placebo plus WCM in terms of **(A)** PDQ-39, **(B)** NMSS, **(C)** PDSS, and **(D)** HAMD. (HM, herbal medicine; WCM, western conventional medicine; PDQ-39, Parkinson's Disease Questionnaire-39; NMSS, Non-Motor Symptoms Scale; PDSS, Parkinson's Disease Sleep Scale; HAMD, Hamilton depression rating scale).

**Quality of Life:** Compared with the placebo, meta-analysis of 4 studies (Kum et al., 2011; Wen et al., 2015; Li et al., 2016; Yang, 2017) showed that HM paratherapy had no significance for improving PDQ-39 (WMD: −4.65, 95% CI: −10.97 to 1.68, *P* = 0.15; heterogeneity: Chi^2^ = 19.19, *P* = 0.0002, *I*^2^ = 84%). A trial (Li et al., 2016) had imbalanced baseline comparing HM with placebo. After removing the trial, meta-analysis of 3 studies (Kum et al., 2011; Wen et al., 2015; Yang, 2017) showed that HM paratherapy was superior to the placebo (WMD: −7.65, 95% CI: −11.46 to −3.83, *p* < 0.0001; heterogeneity: Chi^2^ = 0.12, *P* = 0.94, *I*^2^ = 0%; Figure 4A). One randomized controlled trial (RCT) (Cai et al., 2017) showed HM paratherapy was a significant superiority to placebo according to PDQL (*P* < 0.01).

**NMSQuest and NMSS:** One trial (Cai et al., 2017) showed that HM paratherapy produced greater reduction in NMSQuest score than that of placebo (*P* < 0.01). Meta-analysis of 2 studies (Yu, 2016; Yang, 2017) showed that HM paratherapy was favor of NMSS compared with placebo (WMD: −9.19, 95% CI: −13.11 to −5.28, *P* < 0.00001; heterogeneity: Chi^2^ = 0.56, *P* = 0.45, *I*^2^ = 0%; Figure 4B).

**PDSS:** Compared with the placebo, meta-analysis of 3 studies (Pan et al., 2013; Li et al., 2016; Cai et al., 2017) showed that HM paratherapy had no significance for improving PDSS (WMD: 7.10, 95% CI: −2.26 to 16.45, *P* = 0.14; heterogeneity: Chi^2^ = 101.02, *P* = 0.14, *I*^2^ = 98%). A trial (Li et al., 2016) had imbalanced baseline comparing HM with placebo. After removing the trial, meta-analysis of 2 studies (Pan et al., 2013; Cai et al., 2017) showed that HM paratherapy was superior to the placebo (WMD: 10.69, 95% CI: 8.86 to 12.53, *P* < 0.00001; heterogeneity: Chi^2^ = 0.48, *P* = 0.49, *I*^2^ = 0%; Figure 4C).

**HAMD:** Meta-analysis of 2 studies (Guo et al., 2014; Cai et al., 2017) showed that HM paratherapy was superior to the placebo according to HAMD (WMD: −5.87, 95% CI: −7.06 to −4.68, *p* < 0.00001) with mild heterogeneity (Chi^2^ = 1.51, *P* = 0.22, *I*^2^ = 34%; Figure 4D).

### Adverse events

#### HM monotherapy vs. placebo

In the only one study (Zhao et al., 2009), neither the experimental group nor the control group provide any information about adverse events.

#### HM plus WCM vs. placebo plus WCM

**Figure 5:**
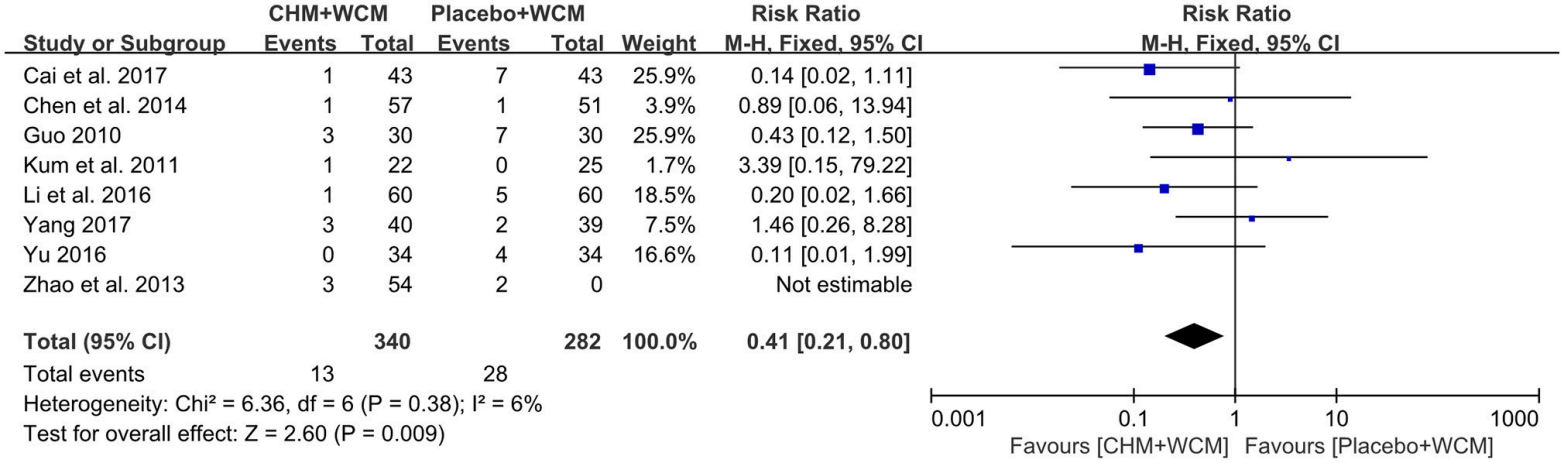
Forest plot of HM plus WCM vs. placebo plus WCM in terms of adverse events.

Eleven RCTs (Guo, 2010; Kum et al., 2011; Pan et al., 2011, 2013; Zhao et al., 2013; Chen M. Y. et al., 2014; Guo et al., 2014; Li et al., 2016; Yu, 2016; Cai et al., 2017; Yang, 2017) reported adverse events, among them 3 studies (Pan et al., 2011, 2013; Guo et al., 2014) reported no adverse events. However, the other 3 studies did not provide any information on adverse event (Pan et al., 2009; Zhao et al., 2009; Wen et al., 2015). Meta-analysis of 8 studies (Guo, 2010; Kum et al., 2011; Zhao et al., 2013; Chen M. Y. et al., 2014; Li et al., 2016; Yu, 2016; Cai et al., 2017; Yang, 2017) showed that HM paratherapy was significant benefit in reducing adverse events compared with control group (RR: 0.41, 95% CI: 0.21 to 0.80, *P* = 0.009; heterogeneity: Chi^2^ = 6.36, *P* = 0.38, *I*^2^ = 6%; Figure 5). The most reported adverse events were gastrointestinal symptoms, such as nausea, vomiting, diarrhea, and abdominal distention in both the HM groups and placebo groups. No life-threatening adverse event was noted in all studies.

### Publication bias

We did not performed the Funnel plot and Egger's test because the number of studies in each meta-analysis was less than ten.

## Discussion

### Summary of evidence

This is first systematic review of randomized double-blind placebo-controlled clinical trials to assess the efficacy and safety of HM formulas for PD. Fourteen high-quality randomized controlled trials (Pan et al., 2009, 2011, 2013; Zhao et al., 2009, 2013; Guo, 2010; Kum et al., 2011; Chen M. Y. et al., 2014; Guo et al., 2014; Wen et al., 2015; Li et al., 2016; Yu, 2016; Cai et al., 2017; Yang, 2017) involving 1,316 patients suffering from PD were identified. HM paratherapy was significant for improving motor symptoms and non-motor functions, whereas there was a negative result of complications of treatment. One trail (Zhao et al., 2009) indicated that HM monotherapy was not superior to the placebo. Eleven out of fourteen studies (Guo, 2010; Kum et al., 2011; Pan et al., 2011, 2013; Zhao et al., 2013; Chen M. Y. et al., 2014; Guo et al., 2014; Li et al., 2016; Yu, 2016; Cai et al., 2017; Yang, 2017) reported no serious adverse events relevant with HM formulas, indicating that HM formulas were generally safe and well tolerated for PD patients. Thus, the findings of present study supported the complementary use of HM paratherapy for PD patients, whereas HM monotherapy for PD is still lack of evidence.

## Limitations

First, the members of the International Committee of Medical Journal Editors published a statement requiring that all clinical trials must be registered in order to be considered for publication (DeAngelis et al., 2004). However, most of included studies didn't formally register. Protocols were not available to confirm free of selective reporting. Thus, further clinical trials must register prospectively in international clinical trials registry platform. Second, although we included randomized double-blind placebo-controlled trials, some inherent and methodological weaknesses still existed in the primary studies: (1) An adequate sample size is crucial to the design of RCTs (Lewis, 1999), but only 4 trials (Kum et al., 2011; Zhao et al., 2013; Li et al., 2016; Yu, 2016) applied pre-trial sample size estimation; (2) PD is a chronic degenerative disease. Long-term efficacy and safety are important assessments to decide the clinical usefulness of an agent in treatment, but only one trial (Li et al., 2016) had the long-term duration of follow-up at 6 month; (3) Intention-to-treat (ITT) analysis could avoid bias and false-positive results, which is the recommended standard approach to analyse data from RCTs (Abraha et al., 2017). However, only two studies (Kum et al., 2011; Li et al., 2016) adopted ITT analysis. (4) In the present study, only 6 trials conducted assessor blinding. Considering the characteristics of outcome measurement of PD patients (e.g., UPDRS), assessor blinding successfully eliminates assessment bias and increases the accuracy and objectivity of outcomes results. Triple blindness is needed in further PD trials. Thus, CONSORT 2010 statement (Moher et al., 2010a) and CONSORT Extension for Chinese Herbal Medicine Formulas 2017 (Cheng et al., 2017) should be applied in trial reporting and publication. Third, the herbal composition, drug formulation and dose of the intervention were not exact same, which would lead to clinical heterogeneity. To assess the efficacy and safety of HMs in a clinical trial, all subjects should be given exactly the same intervention in terms of product identity, purity, dosage, and formulation. Fourth, our study only included trials published in the English and Chinese languages and all the included studies were conducted in China, which may affect the generalizability of present findings. In the further studies, the international coliaboration is needed in order to get more qualified stuidies. Finally, different types and stages of PD can influence disease progression and response to treatment (Reinoso et al., 2015). It is difficult to differentiate the effectiveness of HM formulas targeting these subgroups due to insufficient data of primary studies. The pertinent research should be conducted in future clinical trials, which would contribute significantly to explore the responsiveness of specific PD subgroup to interventions.

### Implications

Up to now, several systematic reviews of traditional medicine for PD (Kim et al., 2012; Wang et al., 2012; Zhang et al., 2014, 2015) have been performed. However, low-quality of included primary studies hindered our conclusions. For example, the two articles written by Kim et al. (2012) and Zhang et al. (2015) belong to high-quality systematic reviews; however, the inherent limitations existed in the included low-quality primary studies. The present study only included randomized double-blind placebo-controlled trials, which remains the gold standard of trial design (Athauda and Foltynie, 2016). These trials reported the detailed randomized methods; placebo-controlled group accounts for the placebo effects that don't depend on the treatment itself (Chen et al., 2017), and thus increasing the reliability of experiment results. The present study provided the evidence to support HM paratherapy for PD, whereas there is still lack of available evidence for HM monotherapy for PD. However, it should be remembered that a lack of scientific evidence does not necessarily mean that the treatment is ineffective (Kotsirilos, 2005). To explore the efficacy of HM monotherapy for PD is needed in the future.

Currently, most available PD therapies are mainly aimed at motor symptoms (Fox et al., 2018). Non-motor symptoms (NMS) are common in PD patients across all disease stages and are a key determinant of QOL (Martinez-Martin et al., 2012). However, NMS have received limited attention and targeted treatments remain a challenge (Kulisevsky et al., 2018). The present systematic review provided the sportive evidence for the effectiveness and safety of HM paratherapy for NMS of PD patients. Thus, it is worthy of further studies.

Although the exact pathogenic mechanisms underlying selective dopaminergic neurons loss in PD remain unknown, it is believed that oxidative stress and mitochondrial dysfunction, protein misfolding and aggregation, inflammation, and apoptotic cell death play central roles in PD pathogenesis (Sarkar et al., 2016). Obviously, PD is not a result of dysfunction of one specific pathway but rather a combination of interconnected events (Lim and Zhang, 2013). The urgent need in PD is the development of neuroprotective therapy targeting more potential signal pathways (Kalia et al., 2015). However, clinical neuroprotective effects of current agents in PD remain unproven (Löhle and Reichmann, 2010). The most frequently used herbs of HM formulas were selected in the present study, including Radix Salviae Miltiorrhizae, Radix Paeoniae Alba, Ramulus Uncariae Cum Uncis, Radix Rehmanniae, Herba Cistanches, Radix Polygoni Multiflori, Rhizoma Ligustici Chuanxiong, Fructus Corni, Radix Angelicae Sinensis, Rhizoma Acori Tatarinowii, and Radix Astragali seu Hedysari. Based on the high-frequency used herbs, the anti-PD mechanisms of the main active ingredients of herbs *in vivo or in vitro* trails are as follows: (1) Antioxidant: Danshensu (from *Radix Salviae Miltiorrhizae*), catalpol (from *Radix Rehmanniae*), 2,3,5,4′-Tetrahydroxystilbene-2-O-β-D-Glucoside (TSG) (from *Radix Polygoni Multiflori*), morroniside (from Fructus Corni) and astragaloside IV (AS-IV) (from *Radix Astragali seu Hedysari)* were shown to alleviate oxidative stress through reducing reactive oxygen species (ROS) level (Bi et al., 2008a; Sun et al., 2011; Chong et al., 2013; Liu et al., 2017; Zhang et al., 2017). Catalpol, and tetramethylpyrazine (TMP) (from Rhizoma Ligustici Chuanxiong) prevented the decrease in the activities of superoxide dismutase, catalase and glutathione peroxidase, and inhibited malondialdehyde overproduction (Bi et al., 2008a; Lu et al., 2014; Li et al., 2016). The Regulation of I3K/Akt/Nrf2 signaling pathway by Danshensu (Chong et al., 2013) and the inhibition of Nrf2/HO-1 pathway by TMP (Michel et al., 2017) contributed to their antioxidant role; (2) Anti-inflammatory: Echinacoside (ECH) (from *Herba Cistanches*) and catalpol showed a stronger inhibition on the productions and/or expressions of several pro-inflammatory cytokine, including nitric oxide (Tian et al., 2006), tumor necrosis factor-α (Tian et al., 2006), interleukin (IL)-1α (Tian et al., 2006), IL-1β and IL-6 (Wang et al., 2015). TMP (Michel et al., 2017) may inhibit the expression of neuroinflammation markers: nuclear factor κB (NF-κB), inducible nitric oxide synthase, cyclooxygenase-2, and glial fibrillary acidic protein; (3) Anti-apoptotic: ECH, TSG, morroniside, paeoniflorin (PF) (from *Radix Paeoniae Alba*), TMP, n-Butylidenephthalide (BP) (from *Radix Angelicae Sinensis*), or AS-IV exerted anti-apoptotic capacity in different aspects, including suppressing the upregulation of the ratio of Bax/Bcl-2 (Sun et al., 2012; Lu et al., 2014; Liu et al., 2017; Michel et al., 2017), the activation of caspase-3 and caspase-8 (Geng et al., 2007; Sun et al., 2011; Lu et al., 2014; Michel et al., 2017) and the expression of Proapoptotic Gene egl-1 (Fu et al., 2014). TSG (Qin et al., 2011) reduced MPP+-induced apoptotic that mediated via PI3K/Akt signaling pathway; (4) The Regulation of mitochondrial dysfunction: ECH, TSG, PF or catalpol attenuated mitochondrial dysfunction not only by suppressing the decrease of cellular ATP levels (Wang et al., 2015), mitochondrial membrane potential (Bi et al., 2008b; Sun et al., 2011, 2012; Wang et al., 2015), the activity of mitochondrial complex I (Bi et al., 2008b), but also decreasing mitochondrial permeability transition pore opening (Bi et al., 2008b); (5) DA and dopaminergic neuron protection: Danshensu, ECH, BP, TMP, AS- IV or β-asarone (from *Rhizoma Acori Tatarinowii*) could enhance the content of DA as well as its metabolites and reduce dopaminergic neuron degeneration (Geng et al., 2007; Chong et al., 2013; Fu et al., 2014; Lu et al., 2014), and led to a marked increase in Tyrosine hydroxylase expression (Geng et al., 2007; Lu et al., 2014; Zhang et al., 2016; Liu et al., 2017). Furthermore, catalpol (Bi et al., 2008b) was found to be a strong inhibitor of MAO-B, which may weaken the biotransformation of 1-methyl-4-phenyl-1,2,3,6-tetrahydropyridine to 1-methyl-4-phenylpyridinium and the metabolism of DA; (6) Reduce α-synuclein accumulation: α-synuclein (α-syn) is a major component of lewy bodies that plays an important role in the pathogenesis of PD (Rocha et al., 2018). Corynoxine (Chen L. L. et al., 2014) (from *Ramulus Uncariae Cum Uncis*) down regulated α-syn in PC12 cells by inducing autophagy. AS-IV (Liu et al., 2017) inhibited the expression of the α-syn via the p38 MAPK signaling pathway. Furthermore, β-asarone (Zhang et al., 2016) promoted the clearance of α-syn via regulating long non-coding RNA Metastasis associated lung adenocarcinoma transcript 1. Because of their advantage of multi-component, multi-target and multi-pathway, HM formulas have great potential application value in neuroprotection. Furthermore, based on the high-frequency used herbs, we can explore the best formula combination, which also ignite the HM treatment method for PD patients.

## Conclusion

The findings of present study showed that HM paratherapy can effectively improve the motor symptoms and non motor symptoms of PD and is well tolerated for PD patients. Thus, the available evidence supported the complementary use of HM paratherapy for PD patients; however, the question on the efficacy of HM monotherapy in alleviating PD symptoms is still open.

## Author contributions

G-QZ and C-SS: study conception and design; C-SS, H-FZ, Q-QX, Y-HS, YW, YLi, YLin, and G-QZ: acquisition, analysis and/or interpretation of data; G-QZ: final approval and overall responsibility for this published work.

### Conflict of interest statement

The authors declare that the research was conducted in the absence of any commercial or financial relationships that could be construed as a potential conflict of interest.
